# White beam diagnostics using X-ray back-scattering from a CVD diamond vacuum window

**DOI:** 10.1107/S1600577519015340

**Published:** 2020-01-01

**Authors:** Roelof van Silfhout, Daniel Pothin, Thierry Martin

**Affiliations:** a School of Electrical and Electronic Engineering, University of Manchester, Sackville Street, Manchester M13 9PL, UK; b ESRF, CS 40220, 38043 Grenoble, France

**Keywords:** X-ray beam diagnostics, beam position monitoring, transmission white beam monitor

## Abstract

Collecting back-scattered X-rays from vacuum windows using a pinhole X-ray camera provides an efficient and reliable method of measuring the beam shape and position of the white synchrotron beam. In this paper, measurements are presented that were conducted at ESRF beamline ID6 which uses an in-vacuum cryogenically cooled permanent-magnet undulator (CPMU18) and a traditional U32 undulator as its radiation sources, allowing tests to be performed at very high power density levels that were adjusted by changing the gap of the undulators. These measurements show that it is possible to record beam shape and beam position using a simple geometry without having to place any further items in the beam path. With this simple test setup it was possible to record the beam position with a root-mean-square noise figure of 150 nm.

## Abstract 2

White beam diagnostics through pinhole imaging of diffusely scattered radiation are presented.

## 1. Introduction   

With the push for high-brilliance radiation sources and their use in studying microscopic samples, the requirement to simultaneously monitor beam intensity, position and shape with high resolution is evident. So far many different approaches for beam monitoring of monochromatic X-ray beams have been reported in the literature. Most of these methods rely on placing a solid or gas in the beam and recording the scattered X-rays, X-ray induced fluorescence, luminescence, X-ray induced photoelectrons or simply the charge carriers generated in semiconducting materials such as thin silicon or diamond diodes. In principle, most of the methods used for monochromatic radiation should also work for polychromatic or ‘white’ X-ray beams such as those produced by insertion devices or bending magnets. In practice, however, solutions for monitoring white beams have suffered from issues caused by the high power density (>400 W mm^−2^ at 27 m from the source) as produced by modern insertion devices. Thin metal foils such as those used for fluorescence measurements or regular thin semiconductor membranes would simply melt under these conditions even when placed in a cooled support. Silicon carbide and single crystal or polycrystalline diamond are exceptions and will handle the heat load when placed in a water-cooled support. At the ESRF, for example, staff have replaced water-cooled beryllium windows (grade PF60 from Brush Wellman) protected by pyrolytic graphite filters at all insertion device front-ends by polycrystalline diamond windows (without pyrolytic graphite) that were grown using the chemical vapour deposition (CVD) method (Biasci *et al.*, 2002 ▸). When placed in water-cooled mounts, these windows not only handle very high power densities due to their large thermal conductivity but also reduce X-ray wavefront distortions compared with beryllium windows due to their superior surface flatness (Espeso *et al.*, 1998 ▸; Goto *et al.*, 2007 ▸; Yabashi *et al.*, 2014 ▸).

CVD diamond can be used in several ways to provide a measure of the X-ray beam position, and several examples have been published in the literature. For example, transmission-mode diamond X-ray detectors have been shown to work with white beams at moderate power levels. Examples of such devices include both quad (Morse *et al.*, 2007 ▸; Bergonzo *et al.*, 2006 ▸; Schulze-Briese *et al.*, 2001 ▸; Desjardins *et al.*, 2014 ▸; Griesmayer *et al.*, 2016 ▸) and multi-pixel (Komlenok *et al.*, 2016 ▸; Pacilli *et al.*, 2013 ▸) electrode arrangements. The large band gap of diamond renders these sensors insensitive for visible light. The quad diode variant has been commercialized (*e.g.* Dectris, Cividec) for monochromatic applications.

Another popular method of using CVD diamond foils for beam diagnostics is to observe the fluorescent light that is emitted in response to X-ray exposure for foils doped with boron or nitro­gen (Diamond Materials) (Degenhardt *et al.*, 2013 ▸; Takahashi *et al.*, 2016 ▸). Using a standard camera for visible-light imaging combined with simple image-processing tools a user readily has access to key X-ray beam parameters such as beam centre, profile and intensity at a reasonable update rate.

Both above-mentioned methods using CVD diamond foils for beam position monitoring have issues that will limit their use as white beam monitors that remain in the beam path. Transmission-mode devices are only available with limited pixel density (Pacilli *et al.*, 2013 ▸; Zhou *et al.*, 2015 ▸), with the most popular devices being the quad diode variant which has a limited linear range and sensitivity dictated by the beam size. Moreover, their configuration with metal electrodes creates additional wavefront distortions. Recently, devices with graphitic electrodes have been tested that promise to reduce such distortions (Komlenok *et al.*, 2016 ▸). Finally, radiation damage in the intense white beam remains a concern which is typically signalled by a reduced charge collection efficiency of the tested devices. Monitoring of the fluorescent light from CVD diamond screens is a very elegant solution and gives access to full information of the impinging X-ray beam. Unfortunately this method is unsuitable for the high-intensity white X-ray beams. This is due to the reduction of the fluorescent yield after exposure of the CVD diamond material. At beamline ID6, for example, a reduction of fluorescence yield of about 15% was seen after 12 h of exposure to the white beam (T. Martin, private communication).

In this paper we report on a transparent white X-ray beam imaging proof-of-principle experiment that records X-rays back-scattered from standard vacuum windows such as those installed at beamline front-ends to separate the machine vacuum from that of the beamline vacuum. The experiments were carried out at beamline ID6 at the ESRF. This beamline features two undulators in series, one conventional hybrid undulator and a cryo-cooled undulator providing a high-power-density test facility for white beam X-ray beam diagnostics.

## 2. Experimental   

The white beam test chamber is located in the ID6 optics hutch at a distance of approximately 27 m from the source, just downstream of the white beam slits, which allowed us to vary the beam size incident on the 50 µm-thick CVD diamond window (Diamond Materials GmbH, Freiburg, Germany). The window was mounted on a water-cooled copper support creating an 8 mm-diameter aperture for the beam to pass through. Thermal contact between the diamond disc and the water-cooled copper support was by means of a spring-loaded clamping ring covering a 2 mm-wide ring at the perimeter of the 12 mm-diameter window. Although this clamping method does not offer the best heat conducting contact with the water-cooled mount, it did offer a flexible way to exchange the window if needed.

At the time of the experiments, ID6 had two undulators installed, a prototype cryo-cooled CPMU18 and a conventional U32. The CPMU undulator has an 18 mm period (110 periods, 2 m long), is equipped with Nd_2_Fe_14_B magnets (Neorem Magnets, 495T), *B*_r_ = 1.15 T, that were cooled to 150 K, and features a minimum gap of 6 mm. The fundamental of the radiation produced at the smallest gap size is at approximately 10 keV. At the smallest gap setting of 6 mm, the power density at 27 m from the source is approximately 345 W mm^−2^. For the U32 undulator the energy of the fundamental at a gap of 11 mm is 3.8 keV; the maximum power density at 27 m from the source is 125 W mm^−2^. U32 has 50 periods including the end field structure; the total length is 1.6 m. When both undulators are used jointly, a maximal power density of almost 500 W mm^−2^ is reached at the test chamber position.

**Figure 1:**
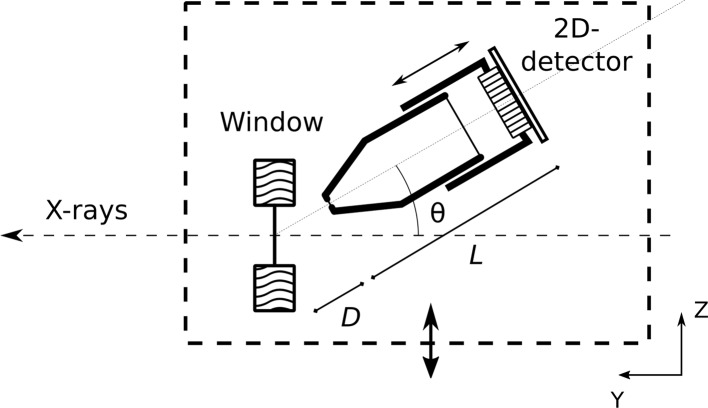
Side view (cross section) of the measurement setup. The white X-ray beam is travelling from right to left (*y*-direction) and traverses a thin CVD diamond window that is held in a water-cooled copper support. A pinhole camera with a variable camera length *L* records the back-scattered X-rays. The pinhole is located at the tip of the conically shaped nosepiece at a distance *D* from the window. The pinhole camera is placed at an angle of 36 (±2)° with the beam path, which is unobstructed. The pinhole camera axis is in the *YZ* plane. The CVD diamond window and pinhole camera setup (dashed box) are placed in a vacuum chamber and can be translated in the *Z*-direction using a remote-controlled stepper motor.

The setup shown in Fig. 1 ▸ was placed in a dedicated vacuum vessel that was located downstream of white beam slits in the optical hutch of ID6. Besides the CVD diamond window assembly our setup includes a pinhole camera in a back-scatter arrangement tilted out of the horizontal (synchrotron) plane by 36 (±2)° such that the white beam could pass unimpeded. The camera itself consisted of a complementary metal-oxide-semiconductor (CMOS) sensor that is fibre-optically coupled to a terbium-doped gadolinium oxysulfide (Gd_2_O_2_S:Tb, Gadox or P43) scintillator. The CMOS sensor consists of 1280 × 1024 pixels, each 7 µm × 7 µm. To make the system insensitive to visible light, a thin layer of aluminium covered the scintillator. Details of the sensor and the method used to fibre-optically couple the CMOS (IBIS-4 ON semiconductor) sensor to the scintillator can be found elsewhere (van Silfhout & Kachatkou, 2008 ▸). The aforementioned work also measured the line spread function of the camera system for X-rays to be 94 µm by exposing the sensor through a 10 µm-wide slit. The camera system was characterized by using the photon transfer curve method (Janesick, 2007 ▸) to give a camera read noise of 1.7 arbitrary digital units (ADU) and a dynamic range of 66.5 dB (Scott *et al.*, 2009 ▸). The fibre optic faceplate consisted of 6 µm-diameter fibres with a fibre-to-core cladding ratio of 19:1 (BXE387-6 from InCom Inc.).

The sensor was read out by a dedicated embedded system that allowed full control over key sensor parameters such as integration time, automatic background subtraction, collection of beam profiles and/or images (Scott *et al.*, 2009 ▸).

Due to the geometry of the existing window mount, the closest distance between pinhole and window that we could achieve was *D* = 20 (±1) mm. The camera length *L* was limited by the size of the vacuum chamber and fixed at 50 (±2) mm; larger pinhole camera magnification factors are simply obtained by reducing *D* and/or increasing *L*.

A motorized stage allowed scanning of the setup through the incident beam in the vertical (*Z*) direction with a precision of about 1 µm. The copper mount for the diamond window was cooled using a recirculating cooler set at room temperature.

Two different pinhole materials were used in our experiments. Very precise laser-cut pinholes with diameters of 0.3 mm and 25 µm were laser cut in 50 µm-thick tungsten foil. These were used in earlier experiments with monochromatic radiation (van Silfhout *et al.*, 2014 ▸; Kachatkou *et al.*, 2013 ▸). We also prepared a tapered pinhole with a smallest diameter of approximately 70 µm cut in a 1 mm-thick lead sheet.

## 3. Experiments   

**Figure 2:**
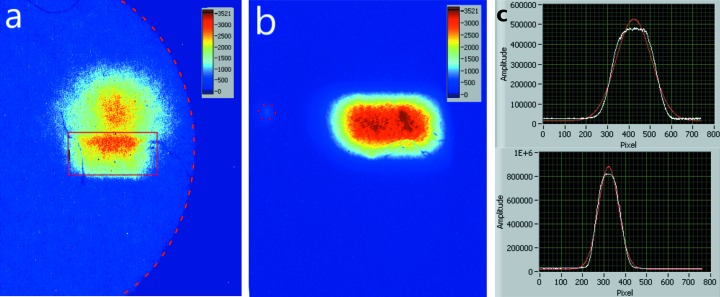
Background-corrected images were taken with a Gadox scintillator fibre-optically coupled CMOS sensor using two different apertures. As source only the U32 undulator is used which is set to a gap of 18 mm. White beam slits were set at 0.5 mm × 0.5 mm. (*a*) White beam image as collected using two 50 µm foils stacked on top of each other, one with a 400 µm-diameter pinhole and one with a 25 µm pinhole (indicated by red box; the two apertures are not perfectly aligned with respect with each other). The area to the right of the red dashed curve is not exposed due to the shadow of the thick copper window mount. (*b*) White beam image as collected using a single 70 µm aperture in a 1 mm-thick Pb foil. Throughout the sensor was oriented in such a way that the horizontal position of the beam was aligned with the vertical position on the sensor and vice versa. Note that the scintillator screen has some scratches and imperfections. The red dashed circled area indicates a feature that is probably due to diffraction from the CVD diamond material. (*c*) Beam profiles (top frame: horizontal beam profile; bottom frame: vertical beam profile) calculated by summing pixel intensities row- and column-wise each for the image in (*b*) with their respective Gaussian best fits.

After alignment of the setup with the white beam using 0.5 mm × 0.5 mm white beam slit settings, pinhole images were taken using both undulators (CPMU18, 6 mm gap, and U32, 13 mm gap) closed. Fig. 2(*a*) ▸ shows the images obtained with a stack of two apertures (one with an aperture of 400 µm and one with an aperture of 25 µm) cut into two foils of 50 µm-thick tungsten. We started off with the single 25 µm aperture but it was simply too transparent to be used with white beam at ID6 and we initially used a stack of the two 50 µm-thick tungsten foils The image from the slit-defined beam due to the 25 µm aperture is highlighted by a red rectangle. The offset of a larger and weaker image is due to the larger 400 µm pinhole which is not well centred with regard to the smaller aperture. The spectrum of the back-scattered X-rays contains a significant amount of photons with energies larger than 30 keV rendering the stack of two 50 µm mm tungsten foils semi-transparent. For comparison we also show an image [Fig. 2(*b*) ▸] taken with a 70 µm aperture cut into a 1 mm-thick lead sheet. It was decided to continue our experiments using the 70 µm aperture in order to obtain images of the incident beam without background or secondary images. Due to the tilt of the pinhole camera axis in the *YZ*-plane the image of the square (0.5 mm × 0.5 mm) beam is reduced in the vertical direction by a factor cos36° as compared with the horizontal direction.

For calibration purposes, images were collected for a series of 0.1 mm steps of the motorized, calibrated in-vacuum height stage on which the setup was mounted. For each image the centre of the beam was determined by fitting a Gaussian distribution to the sum of all the sensor rows. This simple procedure drastically improves the signal-to-noise ratio and provides sub-pixel precision beam positioning capability (Kachatkou & van Silfhout, 2013 ▸).

Even though the measured profile does not always [see Fig. 2(*c*) ▸] resemble a perfect Gaussian, it has been shown that this way of defining the beam position in the presence of noise and background signals results in lower uncertainties compared with methods that rely on centre of gravity or quadrant detection (comparing the intensity over four equal sensor areas) (Scott *et al.*, 2009 ▸). Typical uncertainties resulting from the Gauss best-fit approach for establishing the beam position were 0.89 ± 0.6 µm. During the scan of the vertical position, we also monitor the measured horizontal position of the beam to see whether there is a correlation between the two orthogonal directions. Such a correlation is expected because the setup was not engineered to provide an alignment of the sensor pixel array with a specific plane to within 20–40 µm across the full sensor. Whilst soldering the sensor on the ceramic printed circuit board, for example, tolerances of 10–20 µm are difficult to achieve. Monitoring the horizontal position also would give an indication if, during the calibration scan, the beam moves. The changes observed during the vertical scan correspond to the size of a pixel across the 1 mm range of the full scan.

**Figure 3:**
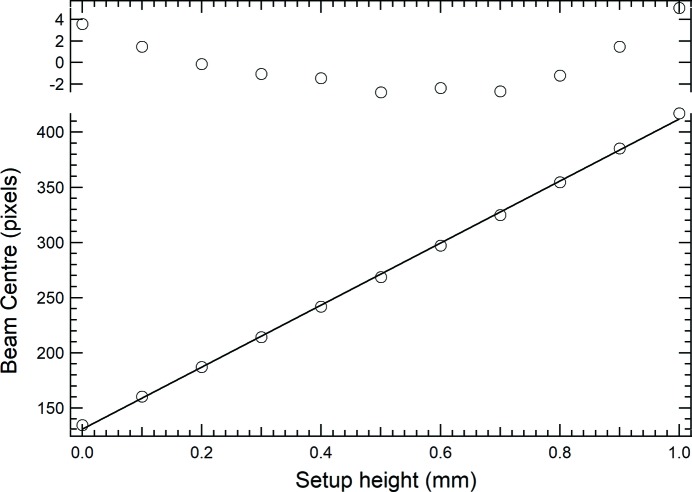
Centre position of the image as recorded for different settings of the vertical translation stage (lower panel). The solid line is a linear fit to the data. The upper panel shows the deviations between the measurements and the best-fit curve in pixel units. The centre position of the beam was determined by fitting of a Gaussian curve to the summed rows and columns of the CMOS image sensor using the Levenberg–Marquardt method. The error bar on the data points is smaller than the markers and is derived from the *R* ^2^ coefficient of determination in the fitting process (Press *et al.*, 2007 ▸). From this scan and the physical camera pixel size of 7 µm × 7 µm, a calibration of 3.57 µm pixel^−1^ is found. The integral non-linearity is 2.1% over a 1 mm range.

From the vertical scans, one of which is shown in Fig. 3 ▸, the calibration for converting the pixel units to distance conversion factor is obtained through the best-fit result of a straight line. The magnification factor was determined from the best fit to be 1.967 (±0.018). Judging from the deviations between the measurements and the best-fit line, the linear dependency needs refinement because the residue has not got a random nature around the zero level.

**Figure 4:**
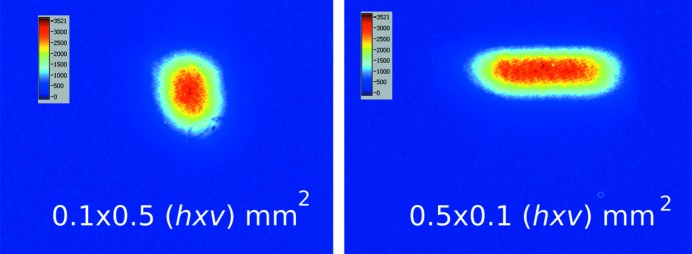
White beam images of the footprint on the CVD diamond window for two slit settings (0.1 mm horizontal and 0.5 mm vertical and vice versa) taken with the pinhole camera setup. The tilt angle of the pinhole camera compresses the image in the vertical direction by a factor of 1/sinθ = 1.7.

Several experiments were performed in order to study the performance of the setup at ID6. For example, the response to changes in white beam slit settings is shown in Fig. 4 ▸. The images were taken with only one undulator (U32, gap size 18 mm) with an exposure time of 0.5 s.

**Figure 5:**
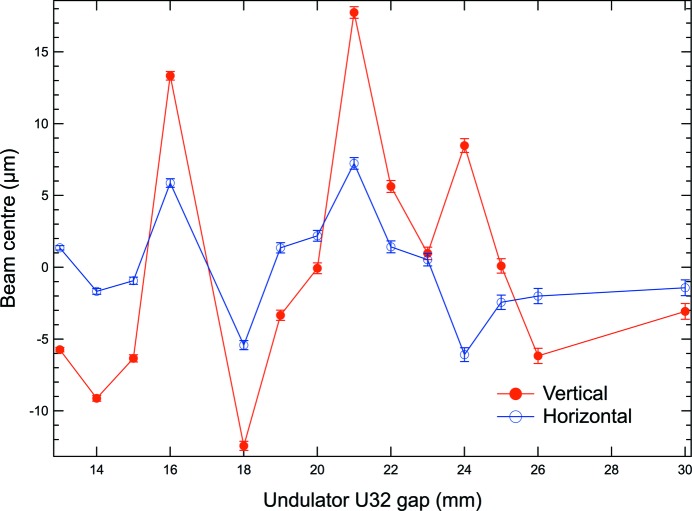
Recording of the beam centre during a scan of the U32 undulator gap.

In order to study the behaviour of the system in response to changes in the setting of the undulator gap a series of measurements of beam position with a fixed slit setting of 0.5 mm × 0.5 mm were conducted (see Fig. 5 ▸). The behaviour of the beam during the scan is of particular interest for spectroscopy applications in which the undulator gap is changed in order to move the undulator fundamental and its harmonics to different energies usually in combination with a rotation of a double-crystal monochromator. A similar series of measurements was carried out for the CPMU18 undulator. These showed a similar behaviour with excursions of the beam centre of 15 µm peak-to-peak. Studies to check whether the contribution of each undulator could be identified were not conducted.

**Figure 6:**
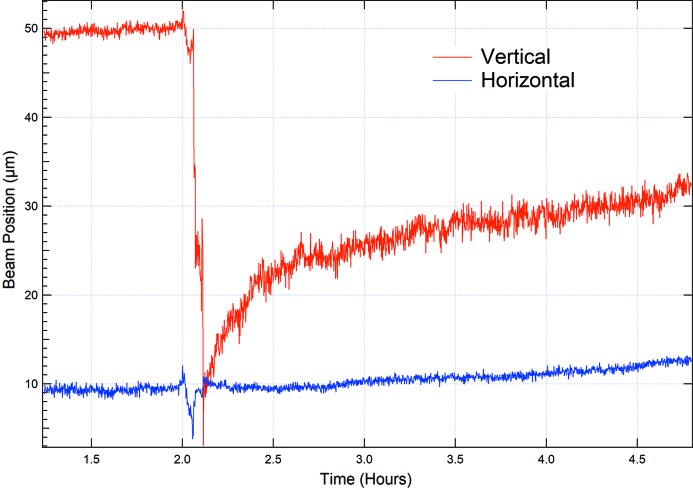
Recording of the beam position as a function of time; a refill event is captured after 2 h of recording. The ESRF synchrotron was operated with a 200 mA current upon refilling without top-up during the time scan.

In order to document the performance of the system in terms of measurement of beam position, we recorded the centre of the beam as a function of time for several hours with both undulators set at their minimum gap. This period included a synchrotron refill in which the machine current increased from 150 to 200 mA over a 10 min filling cycle (see Fig. 6 ▸). Every 0.5 s an image and profile of the beam was collected during this period of measurements. The root-mean-square noise figure determined through statistical analyses from this measurement was 150 nm for the horizontal beam position and 350 nm for the vertical beam position.

## 4. Results, discussion and conclusion   

**Figure 7:**
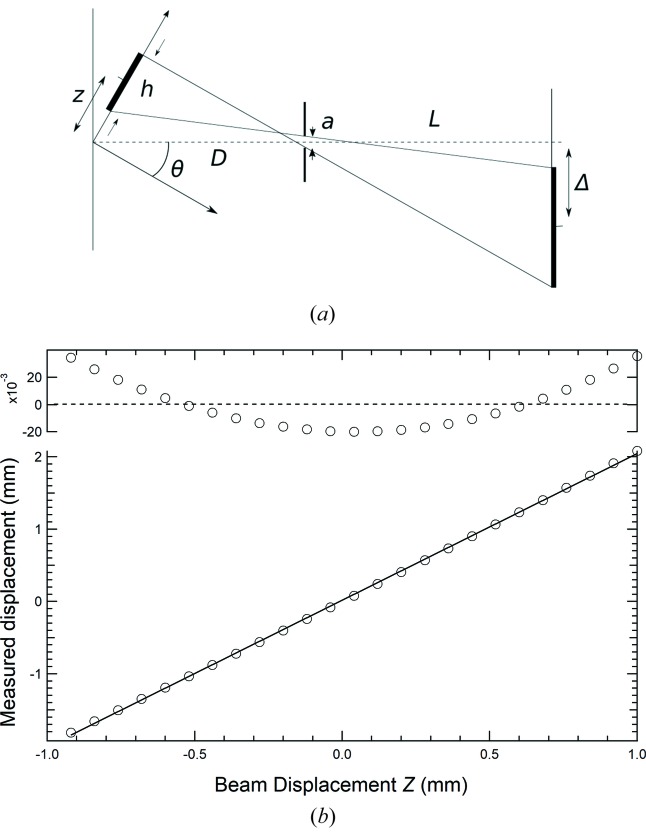
(Top) Geometry of the pinhole camera setup defining the key parameters of the pinhole camera setup. The beam height at the CVD diamond window is given by *h*. The aperture (size *a*) is located between the sensor and window at distances of *L* and *D*, respectively. The axis of the pinhole camera is tilted at an angle with respect to the plane of the window. (Bottom) The expected behaviour (circles) according to equation (1) using the following parameter values: *D* = 20 mm, *L* = 50 mm, *a* = 0.07 mm, *h* = 0.5 mm and θ = 36°. The linear best fit to the calculated points is shown as a solid line through the markers. The data at the top show the deviation from the linear approximation as predicted by equation (1).

The magnification of beam motion in the vertical direction is a function of the tilt angle θ as can be inferred from Fig. 7(*a*) ▸. In order to calculate the expected magnification for our geometry we derive a parametric equation for the shift Δ of the centre of the image corresponding to a shift in the vertical position of the beam of *z* (see Fig. 7 ▸),
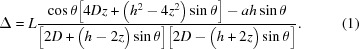
The vertical dimension (*h*) of the incoming beam and aperture size (*a*) have been parameterized; *z* = 0 was taken to be the vertical position of the beam on the window that is aligned with the centre of the pinhole camera axis. Here, we have assumed that the scattering window is infinitesimally thin. Equation (1) has the expected behaviour of 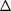 = 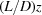 for the case in which the tilt angle *θ* = 0°.

From the calculations based on equation (1) using the parameter values that correspond to the experimental setup it is inferred that the expected magnification should be 2.02. This is to be compared with 1.967 as measured in the calibration of the vertical displacement. A small difference in the geometric parameters (*e.g.* θ = 37.5° and *L* = 49.5 mm) would fully explain this difference between the measurements presented in Fig. 3 ▸ and the simple geometric model shown in Fig. 7 ▸.

**Figure 8:**
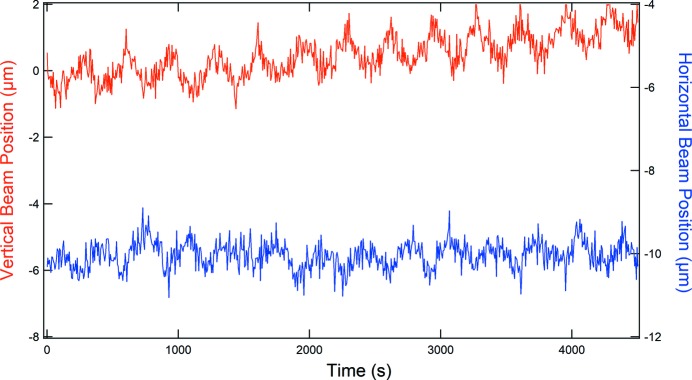
Behaviour of the system with a recirculating bath with a larger temperature variation.

One of the key applications of a white beam diagnostic device is its suitability and stability in recording the position of the beam as a function of time. Early measurements of the beam position as a function of time showed a noticeable oscillation with a peak-to-peak amplitude of 1 µm and a period of 10 min (see Fig. 8 ▸). Such a large-amplitude low-frequency signal is unusual and could not be attributed to synchrotron- or beamline-related issues. At the time of the experiments, the ESRF was not operating in a top-up mode.

Although the specification of the cooling system was unknown, one can infer from the linear expansion coefficient of diamond of 1 × 10^−6^ that excursions on the micrometre scale can be expected for temperature swings of more than 1°C. After changing the recirculating cooler system for another one, which featured a temperature regulation of ±0.1°C precision (Julabo GmbH), these apparent oscillations of the beam position disappeared (see Fig. 6 ▸).

Since the CVD diamond is a polycrystalline material one would expect it to feature diffraction spots that, depending on the incident spectrum, could lead to intense ‘spots’ captured by our setup. Fig. 2(*b*) ▸ captures such an event, signalled by a relatively weak intensity spot that is located to the left of the beam image (indicated by a dashed red circle). Such features have an insignificant contribution in the determination of the beam centre because of their relative low intensity and small area on the detector when compared with the normal slit sizes used during experiments.

In the time domain the system was limited by the bandwidth of the 100 Mbit ethernet link for recording images that was used. For recording profiles only the camera system can provide updates at the rate of up to 100 Hz. Faster direct-type detection X-ray camera systems that do away with the Gadox scintillator are readily available which can provide update rates of 2 kHz or more if required (Garcia-Nathan *et al.*, 2017 ▸). For an exposure of 0.5 s, measured peak counts are about 3000 ADU [see Fig. 2(*b*) ▸]. At 2 kHz frame rates one would expect merely 3 ADU per pixel which is insufficient to collect detailed images of the beam. It is, however, sufficient to measure detailed profiles useful for beam localization because one would add up to 1280 sensor columns and 1024 sensor rows to obtain horizontal and vertical beam profiles which will have peak values significantly higher than that of single pixels (van Silfhout *et al.*, 2011 ▸). For typical use as a diagnostic device that is used to keep X-ray optical elements and samples aligned, the update rates offered by our proof-of-principle setup would, in many cases, suffice.

We demonstrated through deliberate scanning of the setup by a distance of 1 mm that it is able to follow significant changes from a set position. This distance is not the limit of beam excursions that can be followed since this is determined by the size of the CMOS sensor and the magnification used.

In concluding, we have described proof-of-principle measurements taken with a white beam diagnostic system that is based on the recording of back-scattered X-rays from a front-end vacuum window. A bespoke solution would make use of a standard CVD diamond window that is mounted in a dedicated vacuum chamber that integrates a fixed or movable (variable *D*) pinhole. By covering the aperture with a thin metal layer such as beryllium a vacuum-tight chamber is obtained. The X-ray camera would then be placed outside the vacuum on a translation stage to adjust the camera length *L*. With the camera outside the vacuum there are no issues with outgassing of camera components and no delicate electrical feedthrough for relaying the camera signals. With the camera placed outside of the vacuum, varying the camera distance *L* using a translation stage readily changes the magnification factor. Placing the camera outside of the vacuum also makes shielding it much easier in order to protect it from the high radiation levels in the optics hutch.

The use of a pinhole camera to diagnose the white beam allows corrections for contributions of nearby bending magnet sources through simple image-processing steps if required. We have shown that beam changes due to undulator gap adjustments can easily be recorded across the full gap range even though the scattered intensity changes by an order of magnitude. Although the system was limited in magnification range, the uncertainty of the measurements was on par with the best alternatives (Muller *et al.*, 2012 ▸; Revesz *et al.*, 2010 ▸). The design of a bespoke system that allows for a higher magnification factor and the use of a modern scientific CMOS sensor which features smaller pixel size, larger dynamic range and lower noise level should bring the positioning uncertainty down. Even in the non-optimized proof-of-principle setup the proposed measurement setup is able to achieve sub-micrometre precision location of the beam centre. High-resolution imaging of the incident beam would benefit from the use of an aperture with a smaller hole compared with the one used in this study.

Unlike the recording of visible fluorescence the recording of scattered X-rays does not suffer from a dose dependency of the scattered yield.
